# Contrast enhancement for portal images by combination of subtraction and reprojection processes for Compton scattering

**DOI:** 10.1002/acm2.12181

**Published:** 2017-09-12

**Authors:** Masatsugu Hariu, Yuhi Suda, Weishan Chang, Atsushi Myojoyama, Hidetoshi Saitoh

**Affiliations:** ^1^ Graduate School of Human Health Sciences Tokyo Metropolitan University Tokyo Japan

**Keywords:** EPID, image contrast, Monte Carlo simulation, portal image, scatter correction

## Abstract

For patient setup of the IGRT technique, various imaging systems are currently available. MV portal imaging is performed in identical geometry with the treatment beam so that the portal image provides accurate geometric information. However, MV imaging suffers from poor image contrast due to larger Compton scatter photons. In this work, an original image processing algorithm is proposed to improve and enhance the image contrast without increasing the imaging dose. Scatter estimation was performed in detail by MC simulation based on patient CT data. In the image processing, scatter photons were eliminated and then they were reprojected as primary photons on the assumption that Compton interaction did not take place. To improve the processing efficiency, the dose spread function within the EPID was investigated and implemented on the developed code. Portal images with and without the proposed image processing were evaluated by the image contrast profile. By the subtraction process, the image contrast was improved but the EPID signal was weakened because 15.2% of the signal was eliminated due to the contribution of scatter photons. Hence, these scatter photons were reprojected in the reprojection process. As a result, the tumor, bronchi, mediastinal space and ribs were observed more clearly than in the original image. It was clarified that image processing with the dose spread functions provides stronger contrast enhancement while maintaining a sufficient signal‐to‐noise ratio. This work shows the feasibility of improving and enhancing the contrast of portal images.

## 1 INTRODUCTION

Image‐guided radiation therapy (IGRT) employs imaging to maximize geometric accuracy and precision during a treatment session. Various systems, e.g., in‐treatment‐room computed tomography (CT‐on‐rails), kilovoltage cone beam CT (kV‐CBCT), portal imaging and megavoltage cone beam CT (MV‐CBCT) are currently available for the IGRT.^1^, ^2^

CT‐on‐rails and kV‐CBCT can provide superior soft tissue contrast and anatomical information while an additional kV X‐ray source and extra detector are required. The coordinates of two isocenters of kV imaging and treatment beam must be adjusted carefully for correct patient repositioning. Hence, quality assurance (QA) is more complex than that for MV imaging.^3^, ^4^MV portal and MV‐CBCT imaging can be performed in identical geometry with the treatment beam so that accurate geometric information can be provided.^5^, ^6^However, MV imaging suffers from poor image contrast due to the lower difference of X‐ray attenuation and larger Compton scattering compared with kV imaging.^7^, ^8^

Scatter correction methods that comprise scatter estimation and compensation have been reported.^9^, ^10^The beam‐scatter‐kernel (BSK) superposition approach is the most promising in the scatter estimation method with respect to the computational efficiency.^11^The BSK is generally obtained using water rather than heterogeneous mediums and consequently it causes over‐ or underestimation of scatter photons.

In this work, the scatter estimation was performed in detail by Monte Carlo (MC) simulation based on patient CT data. Additionally, an original image processing algorithm was proposed for the scatter compensation. In this process, scatter photons were eliminated and reprojected as primary photons on the electronic portal imaging device (EPID). By the combination of the MC simulation and the proposed image processing, improvement and enhancement of the image contrast were attempted without increasing the imaging dose. To assess the feasibility of the image processing, portal images with and without the scatter compensation were compared.

## 2 METHODS

### 2.A The proposed image‐processing algorithm

The original portal image is generated by primary and scatter photons that occur on the EPID. The signal *P*_o_ at the pixel coordinate (*x*, *y*) is the sum of the signals by primary photons *P*_p_(*x*,* y*) and scatter photons *P*_s_(*x*,* y*) as follows:

(1) $$P_{o}\left(x,y\right)=P_{p}\left(x,y\right)+P_{s}\left(x,y\right)$$

To improve the image contrast, scatter photons must be eliminated. It has been reported that the signal of the EPID *P* is proportional to absorbed dose *D* to the scintillator.^12^, ^13^The *P*_s_(*x*,* y*) can be estimated using absorbed doses by primary photons *D*_p_(*x*,* y*) and scatter photons *D*_s_(*x*,* y*) as follows:

(2) $$P_{s}\left(x,y\right)=P_{o}\left(x,y\right)\frac{D_{s}\left(x,y\right)}{D_{p}\left(x,y\right)+D_{s}\left(x,y\right)}$$

*D*_p_ and *D*_s_ can be calculated by using MC simulation in detail. Accordingly, the signal by primary photons *P*_p_(*x*,* y*) is calculated by the subtraction of *P*_s_(*x*,* y*) from *P*_o_(*x*,* y*),

(3) $$P_{p}\left(x,y\right)=P_{o}\left(x,y\right)-P_{s}\left(x,y\right)$$

However, the entire EPID signal is weakened by the subtraction process. To enhance the image contrast without increasing the imaging dose, we propose a means of reusing the scatter photons that were eliminated by the subtraction process. Eliminated scatter photons are made to reproject from scattering points to the EPID as primary photons on the assumption that Compton interaction did not take place. In consideration of the energy difference between the reprojecting photon *hν*_r_ and the scatter photon *hν*_s_, the signal by the reprojecting photon Δ*P*_r_ is estimated by the ratio of absorbed dose by the reprojecting photon Δ*D*(*hν*_*r*_) to that by the scatter photon Δ*D*(*hν*_s_).

(4) $$\Delta P_{r}=\frac{\Delta D(h\nu_{r})}{\Delta D(h\nu_{s})}\Delta P_{s},$$

where Δ*D*(*hν*_*r*_) and Δ*D*(*hν*_s_) are the absorbed dose to the scintillator by a photon with energy *hν*_*r*_ and *hν*_s_ respectively. They can be estimated by MC simulation for each photon. Δ*P*_s_ is the signal by a scatter photon that is calculated as follows:

(5) $$\Delta P_{s}=\frac{\Delta D(h\nu_{s})}{D_{s}}P_{s}$$

Then, the signal *P*_*r*_(*x*,* y*) by *n* reprojecting photons can be calculated by the summation of Δ*P*_*r*_(*x*,* y*),

(6) $$P_{r}\left(x,y\right)=\sum_{i=1}^{n}\Delta P_{r,i}\left(x,y\right)$$

Finally, the signal of the contrast enhanced image *P*_c_(*x*,* y*) is obtained by the sum of *P*_p_(*x*,* y*) and *P*_r_(*x*,* y*).

(7) $$P_{c}\left(x,y\right)=P_{p}\left(x,y\right)+w\times P_{r}\left(x,y\right)$$

where *w* is the weight factor for adjustment of the contrast enhancement.

### 2.B Simulation of absorbed dose to Gd_2_O_2_S:Tb by a photon

**Figure 1:**
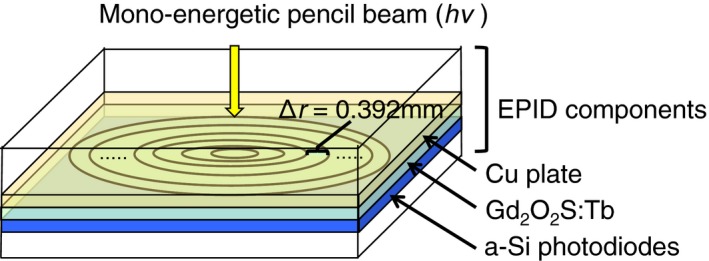
Geometric arrangement of the EPID for the simulation. The EPID is mainly composed of a Cu plate, Gd_2_O_2_S:Tb scintillator and a‐Si photodiodes. In the simulation, equally spaced radial bins with Δ*r *= 0.392 mm (1/2 of pixel width) were arranged, and the absorbed dose to Gd_2_O_2_S:Tb by a photon from the EPID surface was simulated using the DOSRZnrc code.

Figure 1 shows the geometric arrangement of the EPID (Portal Vision a‐S500 on Clinac 21 EX, Varian Medical System) for the simulation. The EPID was modeled in detail according to the design provided by the manufacturer. It is mainly composed of a copper (Cu) plate, terbium‐doped gadolinium oxysulfide (Gd_2_O_2_S:Tb) scintillator and amorphous silicon (a‐Si) photodiodes. The Cu plate filters lower energy photons and electrons; the Cu plate acts as the photons for the electrons converter when high‐energy photons are impinged upon. Then, the scintillator generates fluorescence by electrons from the Cu plate. It is estimated that 99.5% of the total signal is generated within the scintillator.^13^

Electron trajectories are complicated within the EPID. To that end, the absorbed dose to Gd_2_O_2_S:Tb by a photon from the EPID surface was simulated using the DOSRZnrc code,^14^and the dose spread functions of photon energy *hν* and the radial distance from pencil beam *r,* Δ*D*(*hν*,* r*) were obtained. In the simulation, equally spaced radial bins with Δ*r *= 0.392 mm (1/2 of pixel width) were arranged. The EPID consists of not only the main three layers but also low‐density materials, such as air, paper and foamed body. In order to consider the spread of low‐energy particles within low‐density materials, the cut‐off energies of photons and electrons were set to 10 and 521 keV.

### 2.C Acquisition of portal image and 3D‐CT image

The thorax phantom (N‐1 LUNGMAN, Kyoto Kagaku) was modeled as a patient. A water‐equivalent 2 cm* φ* sphere was inserted into the right lung as a tumor. The original portal image was acquired with 6 MV therapeutic beam of a linac (Clinac 21EX, Varian Medical System). The thorax phantom was irradiated with 5 monitor units. The source to the axis distance (SAD) and source to the EPID distance (SDD) were 1000 and 1400 mm, respectively. The field size was set to 40 cm × 30 cm at the EPID, which has 512 × 384 pixels, the pixel size was 0.784 mm × 0.784 mm and the signals were recorded as a 16‐bit integer.

The 3D‐CT data were obtained by the SPECT‐CT scanner (Symbia T2, Seimens Healthcare) with the reconstruction matrix 512 × 512 × 512 and voxel size of 0.7 mm × 0.7 mm × 1.0 mm.

### 2.D Photon sampling and image processing

**Figure 2:**
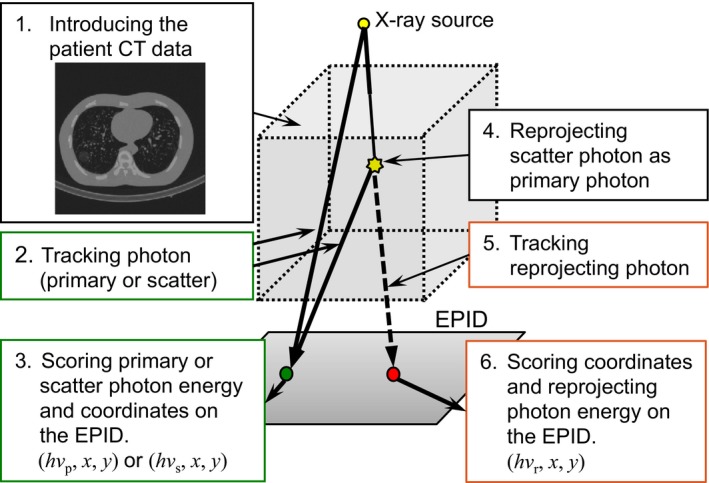
Simplified diagram of the photon sampling for the image processing. The simulation geometry was the same as the MV portal imaging. 3D‐CT data of the thorax phantom was modeled as a patient. A 6 MV beam was reproduced and photon trajectories were investigated in detail. When the photon reached the EPID surface, the coordinates (*x*,* y*) and energy of primary and scatter photons, *hν*_p_ and *hν*_s_, were sampled. If it was a scatter photon, the coordinates (*x*,* y*) and energy *hν*_*r*_ of the reprojecting photon were sampled on the assumption that the Compton interaction does not take place.

3D‐CT data of the thorax phantom was modeled in EGS5 to investigate photon trajectories in detail.^15^ Figure 2 shows a simplified diagram of the photon sampling. The simulation geometry, e.g., SAD, SDD, and field size, was the same as the MV portal imaging described in 2.C. A 6 MV beam was reproduced according to the energy spectrum. When the photon reached the EPID surface, the coordinates (*x*,* y*) and energy *hν* of primary and scatter photons were sampled. Additionally, if it was a scatter photon, the coordinates (*x*, *y*) and energy *hν*_r_ of the reprojecting photon were sampled on the assumption that the Compton interaction does not take place.

To calculate the absorbed dose *D* for each pixel, the deposit energy was sampled within *r* away from the incident point (*x*,* y*) according to the dose spread function Δ*D*(*hν*,* r*). Thus, *D*_s_(*x*,* y*) and *D*_p_(*x*,* y*) for each pixel were calculated by accumulation of the dose spread by scatter and primary photons, respectively. In the subtraction process, *P*_s_(*x*,* y*) and *P*_p_(*x*,* y*) were obtained according to eqs. (2)and (3). In the reprojection process, signals by a scatter photon Δ*P*_s_ were calculated using Δ*D*(*hν*,* r*) and the signal by the reprojecting photon Δ*P*_r_ was calculated by eq. (4) but Δ*D*(*hν*_*r*_)/Δ*D*(*hν*_s_) was replaced with Δ*D*(*hν*_r_, *r*)/Δ*D*(*hν*_s_, *r*) in consideration of the dose spread. The image processing code was developed using the Qt 5.2.1 toolkit and the code was written in C++.

### 2.E Evaluation of portal images with and without the image processing

**Figure 3:**
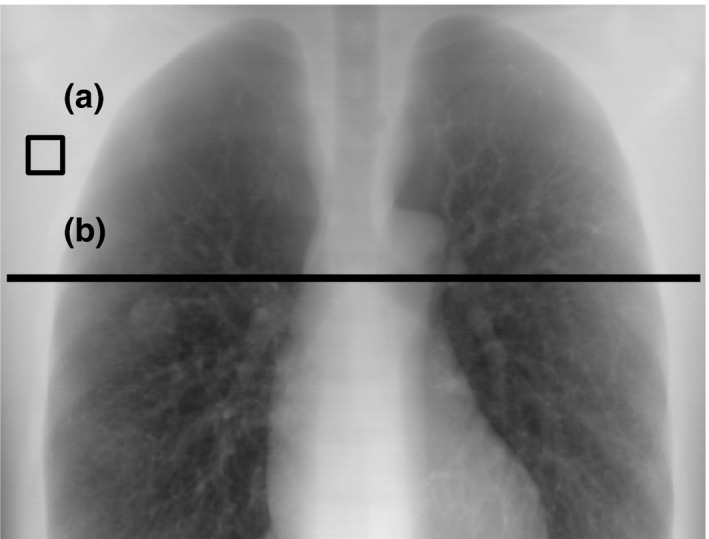
(a) Reference region (square area) and (b) line profile for the image contrast evaluation. The reference region was selected as the homogeneous background in the portal image. The image contrast profile *C*(*x*,* y*) was evaluated along the line profile.

The portal images with and without the proposed image processing were evaluated by the image contrast profile *C*(*x*,* y*) that was used by Kairn et al^16^ *C*(*x*,* y*) was calculated by the following equation:

(8) $$C\left(x,y\right)=\frac{P\left(x,y\right)-P_{\mathrm{ref}}}{P_{\mathrm{ref}}},$$

where *P*_ref_ is the mean signal of the reference region that is indicated as a square in Fig. 3(a). The reference region was selected as the homogeneous background in the portal image. *C*(*x*,* y*) was evaluated along the line profile shown in Fig. 3(b).

## 3 RESULTS

### 3.A Absorbed dose to Gd_2_O_2_S:Tb by a photon

**Figure 4:**
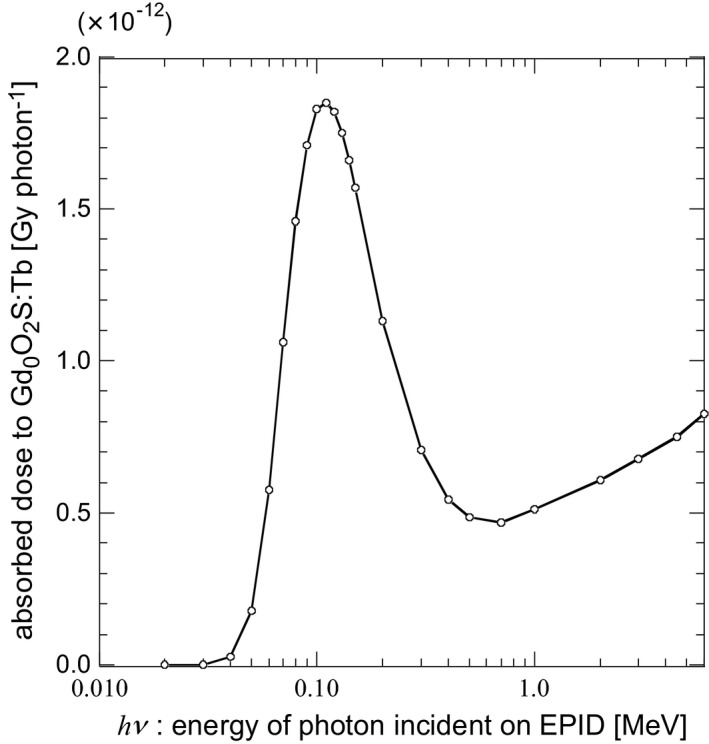
Absorbed dose to the Gd_2_O_2_S:Tb, Δ*D*(*hν*, 0), as a function of photon energy.

Figure 4 shows the absorbed dose to the Gd_2_O_2_S:Tb at *r *=* *0, Δ*D*(*hν*, 0), as a function of photon energy. When *hν* was lower than 0.7 MeV, Δ*D*(*hν*, 0) became maximum at *hν *= 110 keV and decreased quickly with the decrease in *hν*. On the other hand, when *hν* was greater than 0.7 MeV, Δ*D*(*hν*, 0) increased slowly with the increase in *hν*. This tendency suggests that the Cu plate acted as a buildup plate and the large number of recoil electrons reached the Gd_2_O_2_S:Tb.

**Figure 5:**
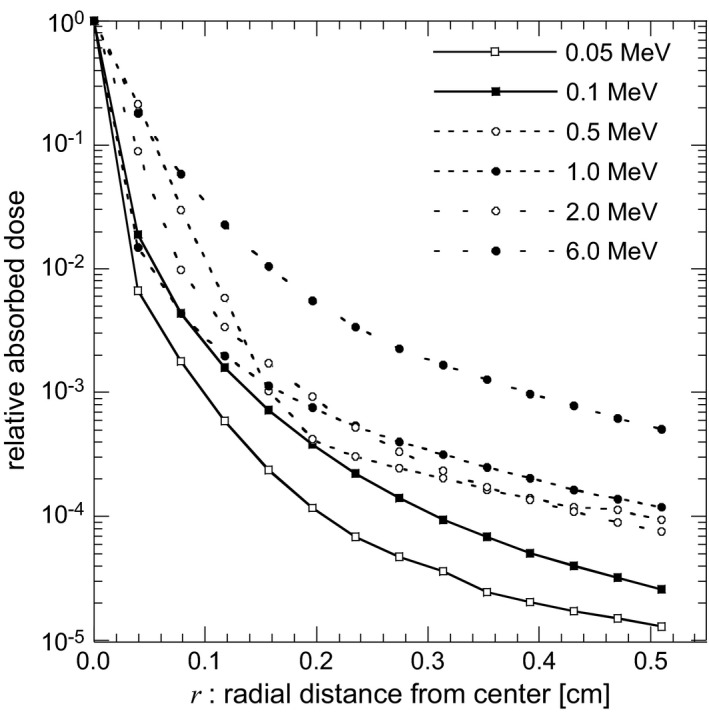
Dose spread functions of Δ*D*(*hν*,* r*) as a function of photon energy and radial distance from pencil beam.

Figure 5 shows dose spread functions Δ*D*(*hν*,* r*) that normalized to absorbed dose at *r *= 0, Δ*D*(*hν*, 0). It was observed that the contribution of the dose spread was increased with the increase in *hν*, and this phenomenon was not negligible in MV imaging. The ratio of Δ*D*(*hν*,* r*) to Δ*D*(*hν*, 0) was lower than 0.5% when *hν* was lower than 1.0 MeV and *r* exceeds 2.5 mm. Accordingly, in the image processing code, Δ*D*(*hν*,* r*) calculations were performed in 0.0 mm ≤ *r *≤* *2.5 mm.

### 3.B Evaluation of portal image with and without the proposed image processing

**Figure 6:**
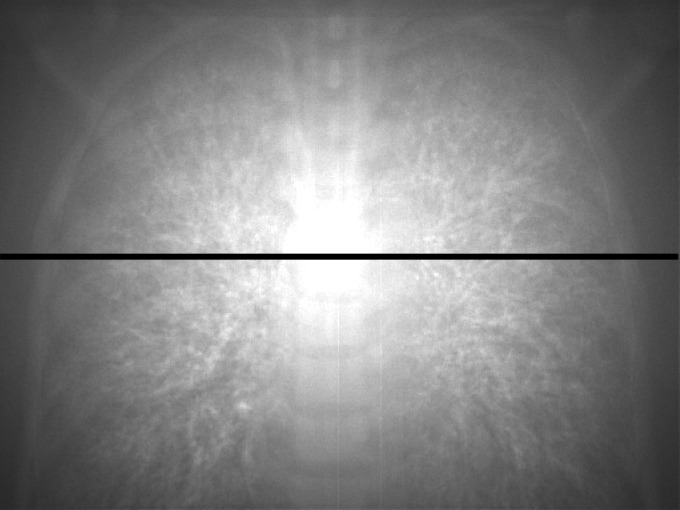
Portal image generated by scatter photons only (*P*_s_ image). Signal profile was obtained along the solid line.

**Figure 7:**
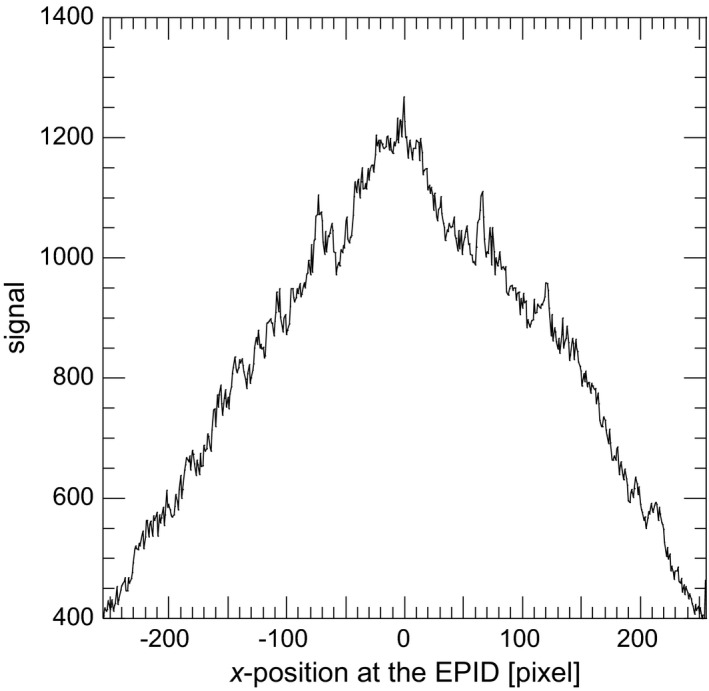
Signal profile along the solid line at the *P*_s_ image.

**Figure 8:**
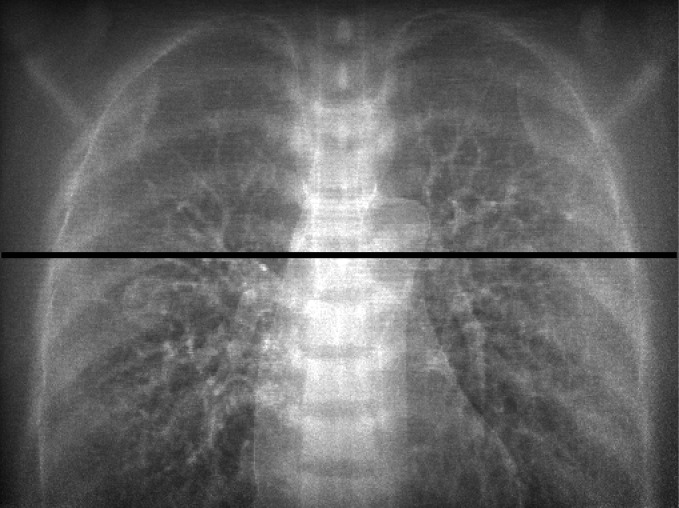
Portal image generated by reprojecting photons only (*P*_*r*_ image). Signal profile was obtained along the solid line.

**Figure 9:**
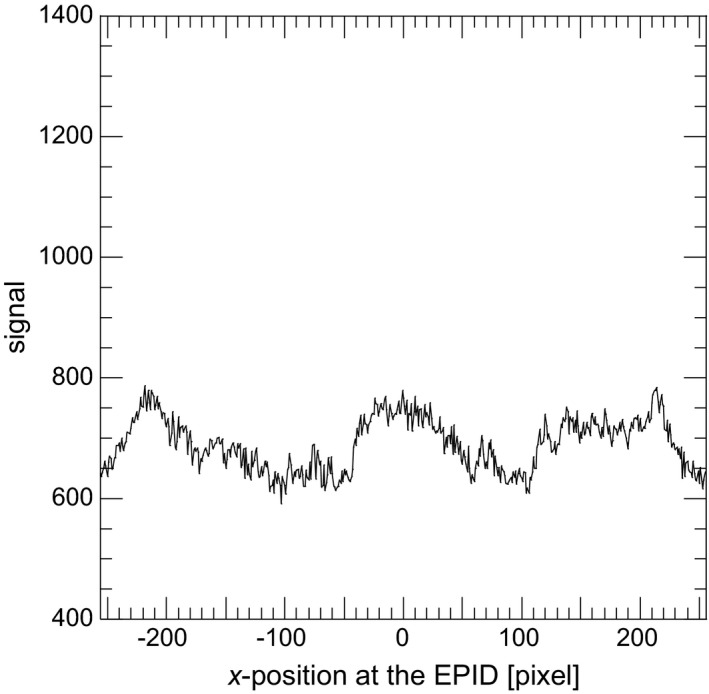
Signal profile along the solid line at the *P*_*r*_ image.

Figure 6 shows the portal image of the thorax phantom by scatter photons only (*P*_s_ image) and Fig. 7 shows the signal profile along the solid line at the *P*_s_ image. The signal by scatter photons was increased near the center of the image. For the 6 MV X‐ray beam, it was clarified that 15.2% of whole EPID signal was generated by scatter photons. Primary photons were mainly scattered in bone structures and the mediastinal space, then the contribution of scattered photons became larger in the center of the EPID. Figure 8 shows the portal image of the thorax phantom by reprojecting photons only (*P*_r_ image) and Fig. 9 shows the signal profile along the solid line at the *P*_r_ image. The convex profile by scatter photons indicated in Fig. 7 was corrected and the thorax structures could be observed by reprojecting photons.

**Figure 10:**
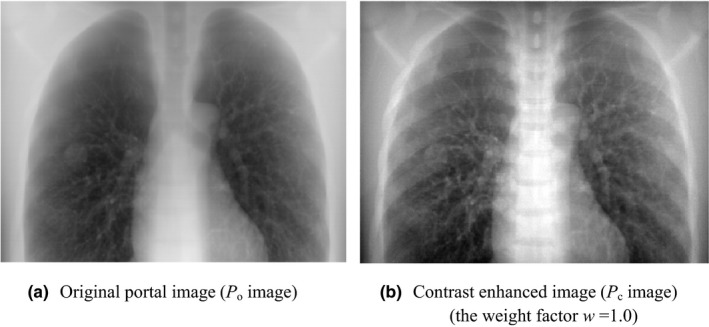
Comparison between original image (*P*_o_ image) and contrast enhanced image (*P*_c_ image). Two images were displayed with the same window width, and gray levels were adjusted to be the same at the coordinates (*x *= 257, *y *= 26) where the spinous process was observed.

**Figure 11:**
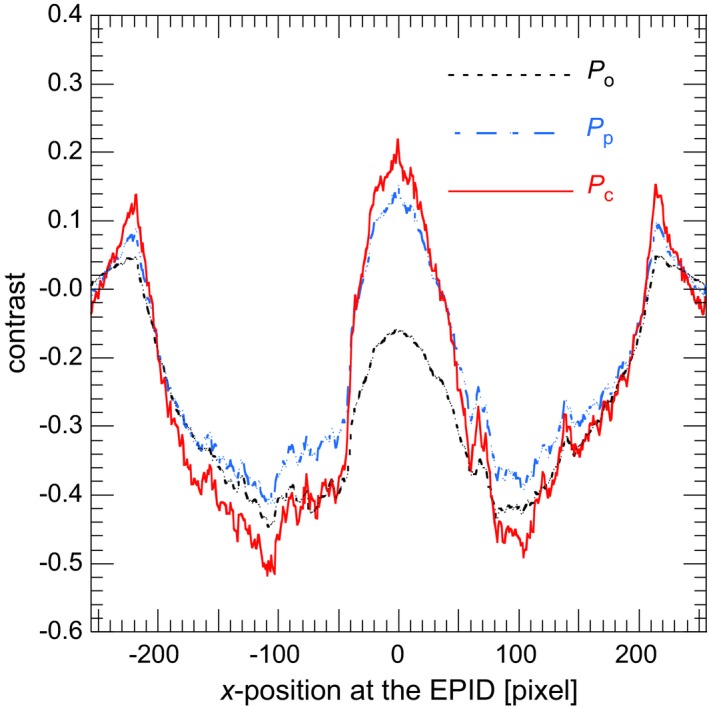
Comparison of contrast profiles between *P*_o,_ *P*_p,_ and *P*_c_ images. The contrast profile was calculated by using the reference region and the line profile as shown in Fig. 3. The dotted line, dashed–dotted line and solid line expressed contrast profiles in *P*_o,_ *P*_p,_ and *P*_c_ images respectively.

Figure 10 shows a comparison between the original portal image (*P*_o_ image) and the contrast enhanced image (*P*_c_ image) that has the weight factor *w *= 1.0. Two images were displayed with the same window width, and gray levels were adjusted to be the same at the coordinates (*x *= 257, *y *= 26) where the spinous process was observed. As a result, the thorax structures, e.g., the tumor, bronchi, mediastinal space and ribs were observed more clearly in the *P*_c_ than in the *P*_o_ image. Figure 11 shows a comparison of contrast profiles between *P*_o_, *P*_p_ and *P*_c_ images. The image contrast of the *P*_c_ image was superior to other images.

## 4 DISCUSSION

Compton interaction becomes dominant above 30 keV for soft tissues and above 60 keV for bone. Within the thorax phantom, most of the interaction was Compton scattering for the 6 MV X‐ray beam. By the MC simulation, it was calcified that 15.2% of the EPID signal was generated by scatter photons. Therefore, it is confirmed that image processing against scatter photons is required for MV imaging.

The proposed image processing was performed by the combination of the subtraction and the reprojection processes. The number of scatter photons increased as the density of the structure increased. Even with the weight factor *w* being 0, namely without the reprojection process, the image contrast was improved. On the other hand, the EPID signal was weakened because 15.2% of the signal was eliminated as the contribution of scatter photons by the subtraction process. Hence, these scatter photons were reprojected as primary photons in the reprojection process. Consequently, it was clarified that scatter photons were utilized as primary photons for more contrast enhancement without increasing the imaging dose.

**Figure 12:**
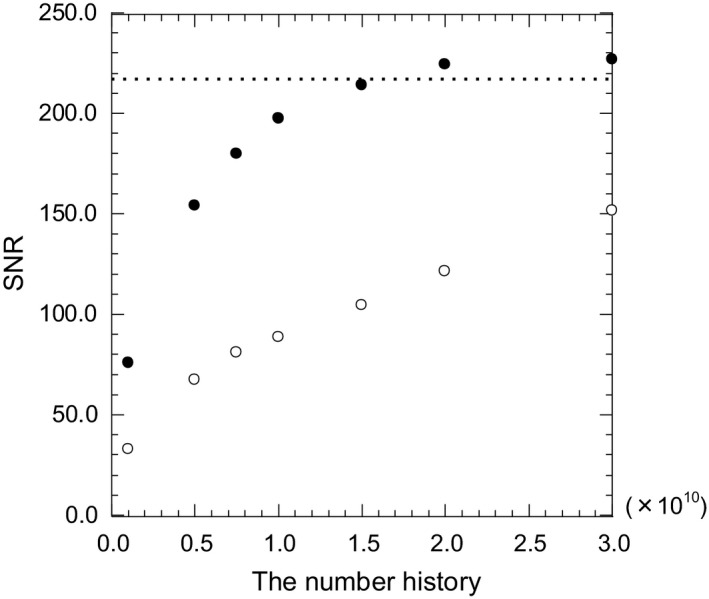
SNR in the reference region of the *P*_c_ image as a function of the number history. Filled and open circles represent the image processing with and without the dose spread functions. The dotted line expresses SNR of the original portal image.

**Figure 13:**
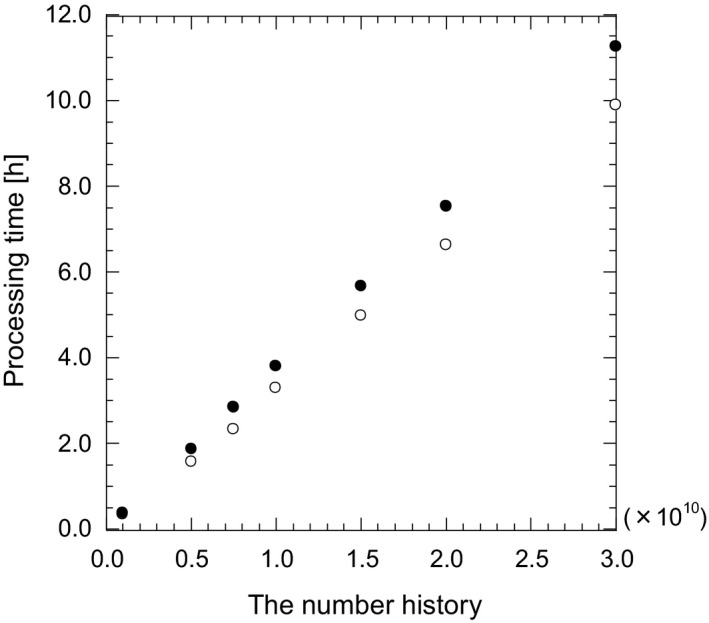
Processing time as a function of the number history. Filled and open circles represent the image processing with and without the dose spread functions.

The proposed image processing includes the photon sampling process using the MC simulation. Thus, the signal‐to‐noise ratio (SNR) of the contrast enhanced image depends on the number history. To improve the photon sampling efficiency, the electron tracking was discarded in this work because the electrons from the thorax phantom reaching the EPID might be disregarded. As a result, the processing time of the photon sampling was shortened by approximately 1/27. The absorbed dose within the EPID was not calculated by the MC simulation but by using the dose spread function. Figure 12 shows SNR as a function of the number history in the reference region of the *P*_c_ image. The SNR of the *P*_c_ image by using the dose spread function reached that of the original portal image when the number history was more than 1.5 × 10^10^. On the other hand, the SNR without the dose spread function was 2/3 in spite of the number history being 3.0 × 10^10^. Figure 13 shows the whole processing time as a function of the number history. The time of processing by using the dose spread function increased less than 10% although the SNR was improved. It was clarified that the image processing with the dose spread functions achieves contrast enhancement while maintaining sufficient SNR.

**Figure 14:**
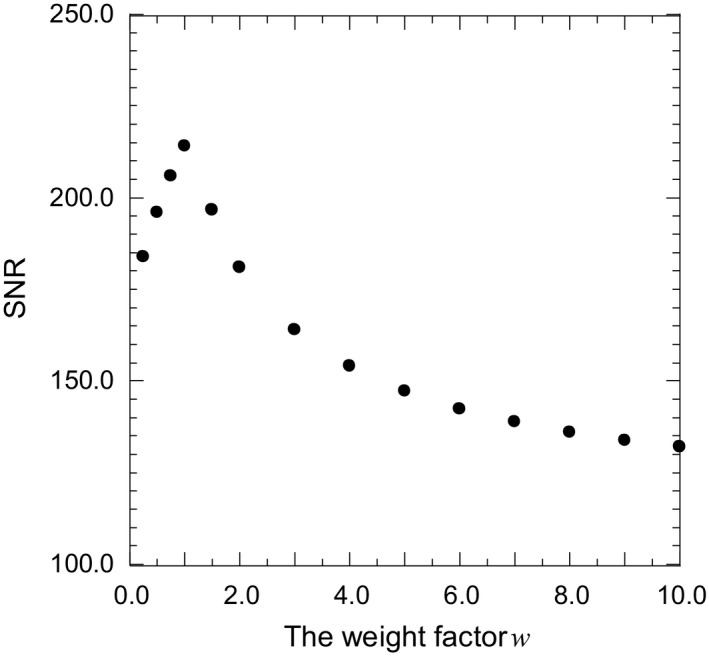
SNR in the reference region of the *P*_c_ image as a function of the weight factor *w*. The SNR increased until *w *=* *1.0, then the SNR decreased with the increase in *w*.

The weight factor *w* amplifies the signal of the reprojecting photons according to eq. (7). On the other hand, there are concerns about the noise enhancement caused by insufficient statistics in MC simulation. Figure 14 shows the SNR as a function of the weight factor *w* in the reference region of the *P*_c_ image. The SNR increased until *w *= 1.0, then SNR decreased with increase in *w*. The SNR is improved by increasing the number history but it takes a longer processing time. Hence, to observe the thorax structures clearly while reducing the processing time, the optimal weight factor and sufficient number history are 1.0 and 1.5 × 10^10^ respectively.

**Figure 15:**
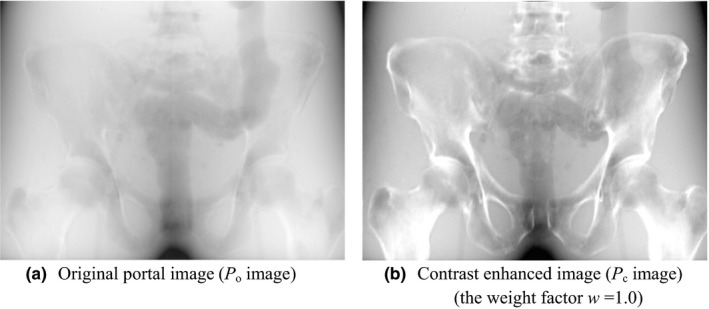
Pelvis phantom images with/without the proposed image processing. Two images were displayed with the same window width, and gray levels were adjusted to be the same at the coordinates (*x *=* *256, *y *=* *20) between vertebral bodies. The contrast enhanced image with sufficient SNR was obtained when the number history was 1.0 × 10^10^.

For other treatment site, the optimal parameters, i.e. the weight factor and the number history, could be different. As an example, Fig. 15 shows portal images of a pelvis phantom (Sectional Lower Torso Phantom, The Phantom Laboratory) with and without the proposed image processing. When the weight factor *w* was 1.0, bone structures were enhanced and a hollow cavity that reproduced the diverticulum and rectum were observed clearly. Since large amount of Compton scatter photons from pelvis were reprojected as primary, sufficient SNR was obtained when the number history was 1.0 × 10^10^. Although the density, location and volume of structures within the pelvis are different from that within the thorax, it was confirmed that contrast enhanced images can be obtained with same weight factor. Therefore, the proposed image processing might be available for major treatment sites with the weight factor *w *= 1.0 and at least 1.5 × 10^10^ histories.

Further works, e.g., speedup of the image processing using the graphics processing units (GPU) based MC simulation,^17^ investigation of optimal parameters considering patient's size and image registration adapting temporal changes in anatomy, are necessary to raise the possibilities and reduce the limitations of the proposed image processing.

## 5 CONCLUSION

Original image processing was proposed to improve and enhance the contrast of portal images. In the image processing, a combination of the subtraction and reprojection processes was performed using the photon sampling data. To improve the processing efficiency, the dose spread functions within the EPID were investigated and implemented on the developed code. In the contrast enhanced image, the structures were observed more clearly than in the original portal image. Consequently, this work demonstrates the feasibility of improving and enhancing the contrast of portal images.

## CONFLICT OF INTEREST

The authors declare no conflict of interest.
